# Can symptoms of anosmia and dysgeusia be diagnostic for COVID‐19?

**DOI:** 10.1002/brb3.1839

**Published:** 2020-09-16

**Authors:** Syeda Anum Zahra, Sashini Iddawela, Kiran Pillai, Rozina Yasmin Choudhury, Amer Harky

**Affiliations:** ^1^ Department of Medicine St George's, University of London London UK; ^2^ Department of Respiratory Medicine University Hospitals Birmingham Birmingham UK; ^3^ Department of Cardiothoracic Surgery Liverpool Heart and Chest Hospital Liverpool UK; ^4^ Department of Integrative Biology, Faculty of Life Sciences University of Liverpool Liverpool UK; ^5^ Liverpool Centre for Cardiovascular Science University of Liverpool Liverpool Heart and Chest Hospital Liverpool UK

**Keywords:** anosmia, COVID‐19, dysgeusia

## Abstract

**Objective:**

Olfactory and taste dysfunction (OTD) is a potential neurological manifestation of coronavirus‐2019 (COVID‐19). We aimed to investigate the diagnostic value of symptoms of anosmia and dysgeusia for COVID‐19.

**Methods:**

A comprehensive electronic search was conducted using PubMed, MEDLINE, Scopus, Cochrane database, and Google Scholar from 1 June 2020 to 12 June 2020. All studies reporting symptoms of anosmia and dysgeusia in COVID‐19‐positive patients were included. A total of 23 studies were included in the systematic review.

**Results:**

Symptoms of anosmia and dysgeusia were frequently reported by COVID‐19‐positive patients. Symptoms were more common in females and in younger patients. There was no direct association between the severity of COVID‐19 and the presence of symptoms. However, some evidence was found for a longer duration of these symptoms and increased severity of COVID‐19 infection in young patients.

**Conclusion:**

OTD is commonly reported by COVID‐19 patients. Due to limited literature on the association between OTD and COVID‐19, it is currently not possible to conclude that these symptoms alone can be used to diagnose COVID‐19. However, the presence of OTD can potentially be used as a screening tool for COVID‐19 especially in young and female patients. Further research is required to establish the true diagnostic value of these symptoms and efficacy as screening tools for COVID‐19 patients.

## Graphical abstract

Neurological manifestations of COVID‐19 can be in any form, assessment of such symptoms should be taken into consideration when examining patients for possible COVID‐19 infection.

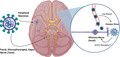

## 1 INTRODUCTION

Coronavirus disease 2019 (COVID‐19) is caused by severe acute respiratory syndrome coronavirus‐2 (SARS‐CoV‐2) (Yuki, Fujiogi, & Koutsogiannaki, 2020). Since the introduction of COVID‐19 to the human population in the Chinese city of Wuhan, it has spread rapidly across the globe and was officially considered a pandemic in March 2020 (World Health Organization, 2020; Yuki et al., 2020). As of 16 August 2020, there have been > 21 million cases with > 700,000 deaths globally (World Health Organisation, 2019). Coronaviruses (CoV) belongs to the *Coronavirinae* subfamily and are known for their microscopic crown‐like appearance (Chen, Liu, & Guo, 2020a; Ren et al., 2020). The COVID‐19 pathogen is a human RNA virus and belongs to the β‐CoVs in the CoV phylogenetic tree (Chen, Liu, et al., 2020). Genome examinations reveal that the novel SARS‐CoV‐2 is 87.99% genetically similar to bats SARS‐like coronavirus and genetically distant from the previously known SARS and MERS viruses (Lovato & Filippis, 2020; Ren et al., 2020). The virus is transmittable from person‐to‐person via respiratory droplets and has a basic reproductive number of approximately 1–3 (Flahault, 2020; Lovato & Filippis, 2020).

Studies report that COVID‐19 patients can present with fever, dry cough, dyspnea, and fatigue (Guan et al., 2020; Lovato & Filippis, 2020). In severe cases, the infection can cause viral pneumonia leading to severe acute respiratory distress syndrome or even death (Guan et al., 2020; Lovato & Filippis, 2020). Symptoms of pharyngodynia, nasal congestion, and rhinorrhoea have also been reported in infected patients (Lovato & Filippis, 2020). Sense of smell is controlled by the olfactory cranial nerve (Cranial & Nerve). The European Rhinology society reported that a significant number of COVID‐19 patients (20%–60%) appear to have loss of smell also known as anosmia (IMPORTANT INFO ON, 2020). Furthermore, reports from China, South Korea, and Italy also reported anosmia in COVID‐19 patients (Entuk.org., 2020; Guan et al., 2020; Kang, Cho, Lee, Kim, & Park, 2020; Lee, Min, Lee, & Kim, 2020). In the UK, anosmia is classed as an official symptom for COVID‐19 (Statement from the UK, 2020). Anosmia may be the first presenting symptom, preceding the occurrence of other COVID‐19 symptoms such as cough and fever (Kang et al., 2020). Interestingly, changes in sense of taste also known as dysgeusia have also been reported in infected patients (Carrillo‐Larco & Altez‐Fernandez, 2020).

The presence of anosmia and dysgeusia may help neurologists and otolaryngologists identify patients with COVID‐19 early, allowing prompt management and infection control procedures to be implemented. The current study aimed to systematically review the current literature on the clinical presentation of COVID‐19, specifically focussing on the symptoms of anosmia and dysgeusia and their diagnostic value in COVID‐19 patients.

## 2 METHODS

### 2.1 Electronic database search

A comprehensive literature search was conducted using PubMed, Medline, Scopus, Cochrane database, and Google Scholar using MeSH words including: “COVID‐19," “Coronavirus 2019,” “Anosmia,” “Dysgeusia.” The systematic review was carried out according to the Preferred Reporting Items for Systematic Reviews and Meta‐analysis (PRISMA) (Moher, Liberati, Tetzlaff, & Altman, 2009). We examined articles and abstracts available in the English language. Literature was screened for original data, and any related references were retrieved and checked manually for other relevant studies.

### 2.2 Inclusion and exclusion criteria

Studies were included if the following criteria were met: (a) Articles were original reports, (b) studies included laboratory‐confirmed COVID‐19 patients, (c) studies reported details of clinical presentation, and (d) the reports were published in the English language. Exclusion criteria included: (a) study design: case report, editorial, letter to the editor, or review; (b) studies reporting symptoms in children/infants.

### 2.3 Data extraction

All studies were screened by two authors independently (KP and SI). Any disagreements were resolved by discussion between the review team members. The extracted data were then cross‐checked by another author (SAZ) to validate its accuracy. Included studies were analyzed to extract all available data and assure eligibility for all patients. Description data of patients including age, sex, clinical symptoms, underlying medical conditions, and outcomes were extracted and recorded for all studies.

### 2.4 Methodological assessment of the studies

Quality Assessment of the included qualitative studies was conducted using the Newcastle‐Ottawa Scale (Table 1) (Wells et al., 2020). The scale was devised specifically to allow quality assessment of the nonrandomized studies included in the systematic review. The scale allows assessment of bias using a star‐based rating system with a maximum score of 9 indicating a low risk of bias and a minimum of 0 indicating the highest risk (Wells et al., 2020). Scores ≥ 7 generally represent a low risk of bias (Wells et al., 2020). The quality of included studies was rated by two of the authors (SAZ and SI).

**Table 1:** Quality assessment of included studies using the Newcastle‐Ottawa Scale

Author	Selection				Comparability	Outcomes			Quality
	Representation of patients with COVID−19	Selection of patients with olfactory and gustatory dysfunction	Ascertainment of exposure	Demonstration that outcome of interest was not present at start of study		Assessment of outcomes	Follow‐up long enough for outcomes to occur	Adequate reporting of outcomes	
Lee et al. (2020)	*	*	*	*	**	*	*	*	Good
Hopkins, Surda, Whitehead, et al. (2020)	*	*			*				Poor
Yan et al. (2020)	*	*		*	*		*	*	Poor
Lechien et al. (2020)	*	*	*	*	*	*	*	*	Poor
Lechien et al. (2020)	*	*	*	*	*	*	*	*	Poor
Lechien et al. (2020)	*	*	*	*	*	*	*	*	Poor
Vaira et al. (2020)	*	*	*	*	*	*	*	*	Poor
Vaira et al. (2020)	*	*	*	*	**	*	*	*	Good
Spinato et al. (2020)	*	*	*	*	*	*	*	*	Poor
Carignan et al. (2020)	*	*	*	*	**	*	*	*	Good
Iravani et al. (2020)		*		*		*			Poor
Boscolo‐Rizzo et al. (2020)		*		*		*			Poor
Giacomelli et al. (2020)	*	*	*	*	*	*	*	*	Poor
Wee et al. (2020)	*	*	*	*	**	*	*	*	Good
Bénézit et al. (2020)	*	*		*	*	*	*	*	Poor
Beltrán‐Corbellini et al. (2020)	*	*	*	*	**	*	*	*	Good
Moein et al. (2020)	*	*	*	*	*	*	*	*	Poor
Klopfenstein et al. (2020)	*	*	*		*	*	*	*	Poor
Kaye et al. (2020)	*	*	*	*	*	*	*	*	Good
Mao et al. (2020)	*	*	*	*	*	**	*	*	Good
Hopkins, Surda, Whitehead, et al. (2020)	*	*			*				Poor
Levinson et al. (2020)	*	*	*	*	*	*		*	Poor
Kosugi et al. (2020)	*	*	*	*	*	*	*	*	Poor

Note

Good quality: 3 or 4 stars (*) in selection domain AND 1 or 2 stars in comparability domain AND 2 or 3 stars in outcome domain; Fair quality: 2 stars in selection domain AND 1 or 2 stars in comparability domain AND 2 or 3 stars in outcome domain; Poor quality: 0 or 1 star in selection domain OR 0 stars in comparability domain OR 0 or 1 stars in outcome domain.

### 2.5 Statistical analysis

It was not possible to do pooled analysis due to high heterogeneity in study design and assessment.

## 3 RESULTS

### 3.1 Retrieving studies

**Figure 1:**
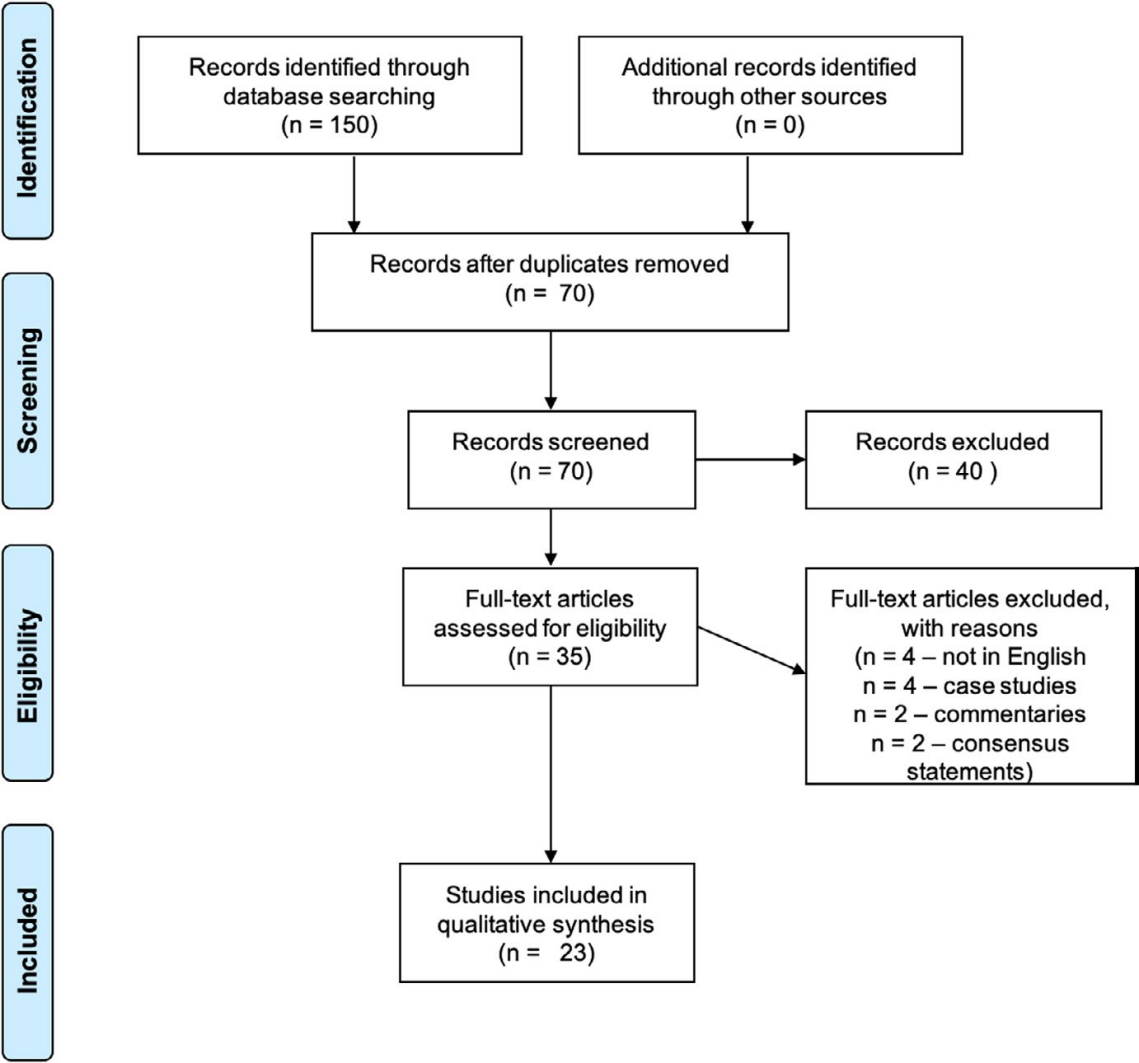
PRISMA flow chart showing the selection process for included studies

A search was started on 10 June 2020, and the last search was on 20 June 2020. A total of 150 articles were retrieved from different databases. After removing duplicates and screening titles and abstracts of studies, we identified 35 studies potentially relevant to the topic. The full text screening of the articles allowed exclusion of 12 studies that did not meet our inclusion criteria. The remaining 23 studies were considered to be eligible for our systematic review. Figure 1 shows a summary of the selection process and criteria.

### 3.2 Assessment of the studies

All 23 studies included were relevant for the review topic. (Beltrán‐Corbellini et al., 2020; Bénézit et al., 2020; Boscolo‐Rizzo et al., 2020; Carignan et al., 2020; Giacomelli et al., 2020; Hopkins, Surda, & Kumar, 2020; Hopkins, Surda, Whitehead, & Kumar, 2020; Iravani et al., 2020; Kaye, Chang, Kazahaya, Brereton, & Denneny, 2020; Klopfenstein et al., 2020; Kosugi et al., 2020; Lechien, Cabaraux, & Chiesa‐Estomba et al., 2020a, 2020b; Lechien et al., 2020; Lee et al., 2020; Levinson et al., 2020; Mao et al., 2020; Moein et al., 2020; Spinato et al., 2020; Vaira et al., 2020; Vaira, Salzano, Deiana, & De Riu, 2020; Wee et al., 2020; Yan, Faraji, Prajapati, Ostrander, & DeConde, 2020) There were no randomized control trials, and studies were mainly retrospective (20) or prospective (3) case‐series, case–control, cohort, cross‐sectional studies (Beltrán‐Corbellini et al., 2020; Bénézit et al., 2020; Boscolo‐Rizzo et al., 2020; Carignan et al., 2020; Giacomelli et al., 2020; Hopkins, Surda, & Kumar, 2020; Hopkins, Surda, Whitehead, et al., 2020; Iravani et al., 2020; Kaye et al., 2020; Klopfenstein et al., 2020; Kosugi et al., 2020; Lechien, Cabaraux, & Chiesa‐Estomba et al., 2020a, 2020b; Lechien et al., 2020; Lee et al., 2020; Levinson et al., 2020; Mao et al., 2020; Moein et al., 2020; Spinato et al., 2020; Vaira, Hopkins, Salzano et al., 2020; Vaira, Salzano, Deiana, & De Riu, 2020, 2020; Wee et al., 2020; Yan et al., 2020). Quality of the studies included in the review was assessed using the Newcastle‐Ottawa Scale (Table 1) (Wells et al., 2020). A “good” quality score required 3 or 4 stars in selection, 1 or 2 stars in comparability, and 2 or 3 stars in outcomes (Wells et al., 2020). A “fair” quality score required 2 stars in selection, 1 or 2 stars in comparability, and 2 or 3 stars in outcomes (Wells et al., 2020). A “poor” quality score reflected 0 or 1 star(s) in selection, or 0 stars in comparability, or 0 or 1 star(s) in outcomes (Wells et al., 2020). Most studies were poor quality, with only 6 studies identified to be of good quality. There is high risk of reporting bias as most studies utilized self‐report questionnaires. These questionnaires are subjective, and it may be possible that some patients exaggerated or over reported their symptoms. Confounders such as the presence of pre‐existing respiratory or otolaryngology disease were not universally accounted for.

### 3.3 Patient background

We included results from 12,314 patients in this review (Table 2) (Beltrán‐Corbellini et al., 2020; Bénézit et al., 2020; Boscolo‐Rizzo et al., 2020; Carignan et al., 2020; Giacomelli et al., 2020; Hopkins, Surda, & Kumar, 2020; Hopkins, Surda, Whitehead, et al., 2020; Iravani et al., 2020; Kaye et al., 2020; Klopfenstein et al., 2020; Kosugi et al., 2020; Lechien, Cabaraux, & Chiesa‐Estomba et al., 2020a, 2020b; Lechien et al., 2020; Lee et al., 2020; Levinson et al.,^2020^;Mao et al., 2020;Moein et al., 2020; Spinato et al., 2020; Vaira et al., 2020, 2020; Wee et al., 2020; Yan et al., 2020). Patient demographics were not reported by all studies but in those that had, most patients were male with the age of the patients ranging from 32 to 60 years (Beltrán‐Corbellini et al., 2020; Carignan et al., 2020; Giacomelli et al., 2020; Hopkins, Surda, Whitehead, et al., 2020; Kaye et al., 2020; Klopfenstein et al., 2020; Kosugi et al., 2020;Lechien, Cabaraux, & Chiesa‐Estomba et al., 2020a,2020b;Lechien et al., 2020; Levinson et al., 2020; Mao et al., 2020; Moein et al., 2020; Spinato et al., 2020; Vaira et al., 2020, 2020; Yan et al., 2020) The presence of comorbidities especially cardiovascular disease, cerebrovascular disease, and diabetes mellitus has been associated with increased risk of COVID‐19 infection and increased severity of the infection. Comorbidities were also not reported by most studies, but in those that had, hypertension (4.7%–28.3%) was the most common disease found in COVID‐19 patients. (Klopfenstein et al., 2020; Kosugi et al., 2020; Lechien, Cabaraux, & Chiesa‐Estomba et al., 2020a, 2020b; Lechien et al., 2020; Levinson et al., 2020; Mao et al., 2020; Moein et al., 2020; Spinato et al., 2020; Yan et al., 2020).

**Table 2:** Studies evaluating symptoms of anosmia and dysgeusia in COVID‐19 patients

Author	Study design	Country	Cohort size	How was anosmia studied?	Patient demographics	Comments
Lee et al. (2020)	Prospective (cohort)	South Korea	3,191	Telephone interview	Age in years: 44 (median) Female gender: 2030% – 63.6% Comorbidities: HTN: 312 (9.8%) DM: 164 (5.1%) Malignancy: 40 (1.3%) ESRF: 1 (0.01%) CHF: 5 (0.2%) CVS: 13 (2.7%)	15.3% (488) reported anosmia or ageusia in early stages and 15.7% (367) reported it in asymptomatic‐to‐mild severity. Prevalence was significantly higher in younger individuals and women. Mean recovery time of anosmia and ageusia was 3 weeks.
Hopkins, Surda, Whitehead, et al. (2020)	Retrospective (Cross‐sectional)	United Kingdom	2,428	Survey distributed online	Does not report on COVID patients directly—only uses those with self‐reported symptoms	17% did not report other COVID−19 symptoms and of those who did, 51% complained of fever or cough. No direct confirmation of COVID−19 test positive, instead relies on the presence of other COVID−19 symptoms
Yan et al. (2020)	Retrospective (Cross‐sectional)	USA	262	Patient‐reported symptoms	Age in years: 40–49 (median) Female: 29 (49.2%) Comorbidities HTN: 8 (13.6%) DM: 5 (8.5%) Cardiac disease: 3 (5.1%) Malignancy: 2 (3.4%)	Smell and taste loss in COVID−19‐positive patients were seen in 68% and 71%, respectively, and impairment in both was independently associated with positive COVID−19 test.
Lechien et al. (2020)	Prospective (Cross‐sectional)	Belgium	78	Identification Test of the “Sniffin Sticks” test	Age in years: 41.7 (mean) Female: 56 (65.1%) Comorbidities HTN: 4 (4.7%) DM: 0 (0) Cardiac disease: 0 (0) Malignancy: not reported CKD: 0 (0)	Patients with initial sudden olfactory anosmia (ISOA) were separated into two groups based on duration of greater or less than 12 days. They were swabbed for COVID−19 and completed psychophysical olfactory evaluation. Complete cohort evaluation was limited by travel restriction but overall in the group with symptoms less than 12 days: 87.5% were positive for COVID−19
Lechien et al. (2020)	Retrospective (case series)	Belgium	86	Subjective assessment using SNOT−22 and impact on life with the sQOD‐NS with further psychophysical evaluation using identification Test of the “Sniffin Sticks” test	Age in years: 36.9 (mean) Female: 263 (63.1%) Comorbidities—precise figures not reported	Total loss of smell was reported by 61.4% in direct contrast to 47.7% identified as anosmic on objective testing. Of 9 anosmic patients to repeat the Sniffin Stick test—5 had improved.
Leichen et al. (2020)	Retrospective (Cross‐sectional)	Europe	417	Self‐report questionnaires—smell and taste section of the National Health and Nutrition Examination Survey and the short version of Questionnaire of Olfactory Disorders‐Negative Statements	Age in years: 36.9 ± 11.4 years Female: 263 (63%) Comorbidities: Hypertension (7%), hyperthyroidism (5%), asthma (7%), allergic rhinitis (15%)	85.6% (356) reported olfactory dysfunction and 88.0% (367) reported gustatory dysfunction. Olfactory dysfunction (OD) appeared before other symptoms in 11.8% (49). Women were significantly more likely to report dysfunction.
Vaira et al. (2020)	Retrospective (case series)	Italy	72	CCCRC test	Age in years: 49.2 (mean) Female—45 (62.5%) Comorbidities—not reported	Olfactory assessment showed 83.3% with hyposmia and 2.8% with anosmia. Gustatory assessment reported hypogeusia in 47.1% and ageusia in 1.4%. No correlation between the presence of OTD and COVID−19 severity.
Vaira et al. (2020)	Retrospective (cohort)	Italy	256	CCCRC test	Age in years: 48.5 (mean) Female—199 (57.7%) Comorbidities—not reported	Multicentre study that conducted objective chemosensitive evaluation, 30% of those who did not report symptoms displayed objective hyposmia, suggesting the prevalence is under reported in questionnaires. No correlation between the presence of OTD and COVID−19 severity.
Spinato et al. (2020)	Retrospective (cross‐sectional)	Italy	202	Telephone interview using SNOT−22 following positive nasopharyngeal and throat swabs	Age in years: median: 56 Female: 105 (52%) Comorbidities Present in 113 (55.9%) patients but—unspecified	OTD alteration was reported in 64.4% (130), of whom 34.6% (30) reported a blocked nose. 11.9% (29) reported change in taste and smell occurred before other symptoms and it was significantly more frequent among women. Using the SNOT−22 criteria, the median symptom level was “severe.”
Carignan et al. (2020)	Retrospective (case–control)	Canada	134	Telephone interview using the Self‐MOQ	Age in years: median age: 57.1 years Female: 70 (52.2%) Comorbidities None reported	Independent presence of anosmia/dysgeusia or both present together were significant predictors of COVID−19 test positivity and may even serve as indicators for testing.
Iravani et al. (2020)	Retrospective (cohort)	Sweden	2,440	Online questionnaire distributed to Swedish population	No reported demographics/symptoms of patients with COVID‐19, study compares prevalence of OTD with other symptoms of COVID‐19	There was a significant difference in odor intensity being reported between patients who had other symptoms of COVID−19 compared to those with no symptoms. This study did not confirm the diagnosis of COVID−19, rather used symptoms that match the clinical picture and had an uncontrolled method of testing smell.
Boscolo‐Rizzo et al. (2020)	Retrospective (cross‐sectional)	Italy	179	Telephone survey of contacts of self‐isolating nonhospitalized COVID−19 patients	No reported demographics/symptoms of patients with COVID‐19, study assess the health of the contacts of said patients	Of the 296 contacts, 175 were not tested and 38.3% (33) reported typical COVID−19 symptoms with 4.0% (17) reporting loss of taste and smell. The prevalence of taste and smell loss in those testing negatives was significantly lower than those who tested positive.
Giacomelli et al. (2020)	Retrospective (cross‐sectional)	Italy	59	Survey of hospitalized patients	Age in years: 60 (median) Female: 19 (32.2%) Comorbidities None reported	34% (20) reported at least 1 olfactory/taste disorder and 18.6% (11) reported both. Taste alterations were more frequently present before hospitalization—but alterations to smell were common in hospital.
Wee et al. (2020)	Retrospective (cross‐sectional)	Singapore	870	Questionnaire presented to patients in the emergency department	Patient demographic data not collected	Presence of olfactory and taste disorder (OTD) had high specificity (98.7%) but low sensitivity (22.7%) as a screening criterion, which is roughly similar to the sensitivity and specificity as history of close contact with COVID−19. Of admitted in‐patients with PCR proven respiratory viruses, COVID−19 patients were significantly more likely to develop OTD.
Bénézit et al. (2020)	Retrospective (cohort)	France—multicentre	259	Web‐based questionnaire	No patient demographic data collected	Hypogeusia and hyposmia, either independently or together, were strongly associated with COVID−19 positivity. Hypogeusia was reported by 24% (63), hyposmia in 20% (51) and both in 17% (43). However, the anonymous nature of the survey meant that accuracy of COVID−19 infection cannot be ratified.
Beltrán‐Corbellini et al. (2020)	Retrospective (case–control	Spain	79	Telephone interview	Age in years: 61.6 (mean) Female: 38 (48%) Comorbidities: none reported	Multicentre study comparing COVID−19‐positive patients with influenza‐positive patients for olfactory and gustatory symptoms. New‐onset olfactory and gustatory symptoms were significantly more common in COVID−19 patients, in those patients who did report symptoms were significantly younger than those who did not.
Moein et al. (2020)	Retrospective (case–control)	Iran	60	USPIT	Age in years: 46.55 (mean) Female: 20 (33.3%) Comorbidities HTN: 6 (10%) DM: 8 (13.3%) Malignancy: 2 (0.03%) None other relevant reported	All but one of the patients had some level of olfactory dysfunction—the mean scoring of the USPIT was 20.98 indicating severe hyposmia. 58% (35) were anosmic and 33% (20) being severely hyposmic.
Klopfenstein et al. (2020)	Retrospective (cohort)	France	114	Unprompted patient reports	Age in years: 54 (mean) Female: 36 (67%) Comorbidities HTN: 7 (13%) CVS: 6 (11%) DM: 2 (4%) Malignancy: 2 (4%)	47% (54) reported anosmia and 85% (46) had dysgeusia. Mean age was 47 and 67% were female. 98% recovered within 28 days—80% had recovered by 14 days.
Kaye et al. (2020)	Prospective (cohort)	USA	237	COVID−19 Anosmia Reporting Tool for Clinicians	Age in years: 36 years (median) Female gender: 129% – 54% Comorbidities: (N/A)	73% (173) of all patients had anosmia prior to diagnosis and was the initial symptom in more than a quarter. 27% (46) reported improvement in anosmia, taking an average of 7.2 days, while 85% improved in 10 days.
Mao et al. (2020)	Retrospective (case‐series)	China	214	Interview	Age in years: 52.7 (mean) Female gender: 127% – 59.3% Comorbidities: 83 had comorbidities: (38.8%) HTN – 51 (23.8%) DM – 30 (14.0%) CVS/CVD – 15 (7%) Malignancy – 13 (6.1%) CKD – 6 (2.8%)	Anosmia/ageusia had no bearing on severity of COVID−19, but were the most common PNS symptoms.
Hopkins, Surda, Whitehead, et al. (2020)	Retrospective (cohort)	UK	382	Online survey	Age in years: 40–49 years (median) Female gender: 74.6% Comorbidities: not reported	Anosmia and hyposmia reported in the majority of completed surveys, with significant improvements seen following one week—complete resolution of olfactory disorders within 11.5% of the 330 reporting issues.
Levinson et al. (2020)	Retrospective (cohort)	Israel	42	Online survey combined with further telephone interview following discharge	Age in years: 34 years (median) Female gender: 19% – 45.2% Comorbidities: 6 patients (9.5%) had comorbidities but unspecified	Of the 35.7% of cohort reporting anosmia, median recovery time was 7.6 days in those that had recovered.
Kosugi et al. (2020)	Retrospective (cohort)	Brazil	253	Online survey	Age in years: 36 (mean) Female gender: 77 (53.1%) Comorbidities: 47 (32.4%) but unspecified	Most of the patients reported sudden anosmia as opposed to hyposmia. However, in patients with hyposmia—this tended to recover sooner.

Abbreviations: CCCRC, Connecticut Chemosensory Clinical Research Centre; CKD, chronic kidney disease; DM, diabetes mellitus; HTN, hypertension; OTD, olfactory and taste dysfunction; Self‐MOQ, Self‐reported Mini Olfactory Questionnaire; SNOT‐22, Sino‐nasal Outcome test; sQOD‐NS, Questionnaire of olfactory disorders—negative statements; USPIT, University of Pennsylvania Smell Identification test.

### 3.4 Prevalence of anosmia and dysgeusia

The prevalence of olfactory and taste dysfunction (OTD) in COVID‐19 varies widely in the literature (Table 2). A large multicentre study from Europe reports over 85% of patients who have been confirmed positive for COVID‐19 report OTD (Lechien et al., 2020). This proportion remains consistently high in the absence of nasal obstruction (Hopkins, Surda, & Kumar, 2020). Beltrán‐Corbellini et al. conducted a case–control study comparing the prevalence of olfactory and gustatory dysfunction in patients with COVID‐19 compared to those with other influenza‐like diseases (Beltrán‐Corbellini et al., 2020). The results corroborated that of Leichen et al., with over 80% reporting smell disorders and over 90% reporting taste dysfunction (Lechien, Cabaraux, & Chiesa‐Estomba et al., 2020a). Both symptoms were significantly more likely to occur whether the patient was COVID‐19‐positive. In contrast, a smaller study done by Giamecello et al. sets a smaller prevalence, with 34% of their sample reporting at least one form of taste and smell dysfunction (Giacomelli et al., 2020).

OTD exists on a spectrum, from hyposmia and hypogeusia to anosmia and ageusia. True absence of taste or smell is rarer than dysfunction. Vaira et al. reported the results of a case series of 72 COVID‐19 patients who underwent objective olfactory and gustatory testing. An assessment revealed 83.3% and 47.1% had hyposmia and hypogeusia, respectively. This contrasts with anosmia (2.8%) and ageusia (1.4%) (Vaira et al., 2020). Klopfenstein et al. conducted a retrospective cohort study that set the prevalence of anosmia at 47% and dysgeusia at 85% in its sample (Klopfenstein et al., 2020). It is important to regard nasal obstruction as a possible explanation for OTD. Lechien et al. and Spinato et al. report a minority of patients with concomitant nasal obstruction, but this aspect is not widely accounted for in the literature (Lechien, Cabaraux, & Chiesa‐Estomba et al., 2020b; Spinato et al., 2020). A consistent feature in the evidence base is that OTD is significantly more common in younger patients and those who are female. (Klopfenstein et al., 2020; Lechien, Cabaraux, & Chiesa‐Estomba et al., 2020b; Spinato et al., 2020; Vaira et al., 2020)

### 3.5 Other symptoms

In most studies that reported olfactory or gustatory dysfunction, a range of other symptoms such as fever, cough, headache, fatigue, and arthralgia/myalgia were also reported (Table 3) (Carignan et al., 2020; Giacomelli et al., 2020; Klopfenstein et al., 2020; Kosugi et al., 2020; Lechien, Cabaraux, & Chiesa‐Estomba et al., 2020a, 2020b; Lechien et al., 2020; Mao et al., 2020; Moein et al., 2020; Spinato et al., 2020; Vaira et al., 2020; Wee et al., 2020; Yan et al., 2020). The most commonly reported symptom in the majority of the studies was fever with a few reporting cough to be the most common. In contrast, Yan et al. reported fatigue as the most common symptom with 81.4% of patients with the symptom (Yan et al., 2020) This is consistent with Klopenstein et al. which reported 93% of COVID‐19 patients had fatigue (Klopfenstein et al., 2020; Yan et al., 2020). Interestingly, studies have also reported anorexia as a symptom. (Carignan et al., 2020; Kosugi et al., 2020; Mao et al., 2020; Moein et al., 2020; Spinato et al., 2020) The presence of anorexia varies between 3% and 56% (Carignan et al., 2020; Kosugi et al., 2020; Mao et al., 2020; Moein et al., 2020; Spinato et al., 2020). It is important to note that dysfunction in a sense of taste or smell could cause changes in appetite. Therefore, an examination of olfactory and gustatory function may be warranted in patients with anorexia.

**Table 3:** Other COVID‐19 associated symptoms

Author	Study design	Cohort Size	Other Symptoms					
			Fever	Cough	Headache	Arthralgia/Myalgia	Fatigue	Anorexia
Yan et al. (2020)	Retrospective (Cross‐sectional)	262	41 (69.5%)	39 (66.1%)	25 (42.4%)	37 (62.7%)	48 (81.4%)	‐
Leichien et al. (2020)	Prospective (Cross‐sectional)	78	62 (72.9%)	42 (48.6%)	52 (60.0%)	36 (42.9%)	‐	‐
Leichien et al. (2020)	Retrospective (Cross‐sectional)	417	48%	78%	45%	31%	‐	‐
Vaira et al. (2020)	Retrospective (case series)	72	69 (95.8%)	60 (83.3%)	30 (41.6%)	‐	48 (66.7%)	‐
Spinato et al. (2020)	Retrospective (cross‐sectional)	202	113 (55.9%)	122 (60.4%)	86 (42.6%)	90 (44.6%)	‐	110 (54.5%)
Carignan et al. (2020)	Retrospective (case–control)	134	50 (37.3%)	90 (72.4%)	87 (64.9%)	76 (56.7%)	‐	75 (56.0%)
Giacomelli et al. (2020)	Retrospective (cross‐sectional)	59	43 (72.8%)	22 (37.3%)	2 (3.4%)	3 (5.1%)	‐	‐
Wee et al. (2020)	Retrospective (cross‐sectional)	870	21 (60%)	10 (28.5%)	‐	‐	‐	‐
Moein et al. (2020)	Retrospective (case–control)	60	46 (77%)	35 (58%)	22 (37%)	5 (8%)	‐	2 (3%)
Klopfenstein et al. (2020)	Retrospective (cohort)	114	40 (74%)	47 (87%)	44 (82%)	40 (74%)	50 (93%)	‐
Mao et al. (2020)	Retrospective (case–control)	214	132 (61.7%)	107 (50%)	28 (13.1%)	‐	‐	68 (31.8%)
Kosugi et al. (2020)	Retrospective (cohort)	253	16 (42.1%)	83 (57.2%)	20(52.6%)	8(21%)	7 (18.4%)	2 (5.2%)

### 3.6 Severity

There is significant variability in the reported severity of symptoms (Table 2). In a cohort of 72 patients, administering the Connecticut Chemosensory Clinical Research Centre Orthonasal Olfaction test (CCCRC) revealed mild to moderate hyposmia as the most common presentation of OTD, while complete anosmia was only present in 2.8% of the cohort. (Vaira et al., 2020) There is similar variability in the presentation of taste dysfunction. In the same cohort, mild to moderate hypogeusia were common while there was only one patient with complete ageusia (complete loss of taste sensation). (Vaira et al., 2020) In another assessment of OTD, a cohort of 283 patients in Italy were assessed using a telephone interview after positive nasopharyngeal and throat swabs. Here, the Sino‐nasal Outcome Test 22 (SNOT‐22) was utilized, and the median score of the 130 patients reporting symptoms was four (Spinato et al., 2020). The SNOT‐22 uses a scale of zero to five, with five indicating the symptoms are “as bad as it can be” and the score of four would indicate the OTD is severe (Hopkins, Gillett, Slack, Lund, & Browne, 2009). Importantly, this study presents an objective assessment of OTD where other studies rely on self‐reported questionnaires.

While there is a variance in the severity of OTD in COVID‐19 patients, there has been research into the presence of OTD as a predictor of severe infection. It should be noted that there is still uncertainty in defining a severe infection; however, numerous proxies have been used in literature. Interestingly, multivariable logistic regression of 169 patients in San Diego showed that the presence of normosmia was independently associated with hospitalization with hyposmia or anosmia 10‐fold less likely to be admitted, suggesting alterations in olfaction are associated with milder infections (Yan et al., 2020). However, further work has failed to recreate the same result. Vaira et al. show that there exists no correlation between the severity of disease and the CCCRC score using pneumonia as a marker of severity (Vaira et al., 2020). This is further highlighted in work by Mao et al., while the most common peripheral nervous system symptoms were OTD, the presence of either smell or taste impairment had no bearing on the severity of disease (Mao et al., 2020).

### 3.7 Recovery

As seen with severity, there is marked variability in the recovery from the OTD (Table 2). In a retrospective analysis of 253 patients with COVID‐19 in Brazil, an online survey indicated that full recovery had a median duration of 12.5 days and with a median follow‐up of 31 days – 121 patients had fully recovered in that time (Kosugi et al., 2020). Similarly, another retrospective analysis of 42 patients in Israel showed a median recovery of 7.6 days in 35.7% of the cohort reporting anosmia (Levinson et al., 2020). However, the follow‐up period was cut short and as such poses a question regarding the true value of recovery. In further studies, the median duration does appear within the range of 1–2 weeks (Lee et al., 2020). Total recovery time among the entire cohort was not well reported among the studies identified; however, Lee et al. noted it took over 3 weeks for a cohort of 3,191 patients in South Korea to fully recover (Lee et al., 2020). The younger population (ages 20–39) were prone to have a longer duration of anosmia than older cohorts (Lee et al., 2020).

### 3.8 Discussion

A large COVID‐19 case series published by the Chinese Centre of Disease Control and prevention reported that among a total of 72, 314 COVID‐19 cases, a total of 22% of patients were identified as suspected cases and 15% were clinically diagnosed based on clinical symptoms (Wu & McGoogan, 2020). The most common symptoms were reported to be fever and cough, with no detailed description of any other symptoms (Wu & McGoogan, 2020). Increasing evidence shows that symptoms of anosmia and dysgeusia are related to COVID‐19 (Lovato & Filippis, 2020; Zayet et al., 2020). In March 2020, American Academy of Otolaryngology‐Head and Neck Surgery suggested that the symptoms of anosmia and dysgeusia should be included in the list of screening tools for COVID‐19 as these symptoms are frequently reported by patients that ultimately test positive for COVID‐19 (AAO‐HNS: Anosmia, Hyposmia). Symptoms of OTD should prompt self‐isolation and testing for COVID‐19. Suspected COVID‐19 cases should be isolated and health personnel in contact should wear appropriate personal protective equipment such as waterproof gowns, gloves, goggles, and surgical masks or FFP2 masks (AAO‐HNS: Anosmia, Hyposmia; Lovato & Filippis, 2020). This systematic review aimed to investigate the diagnostic value of anosmia and dysgeusia for COVID‐19.

Anosmia and dysgeusia were frequently reported in patients who tested positive for COVID‐19 (Beltrán‐Corbellini et al., 2020; Bénézit et al., 2020; Boscolo‐Rizzo et al., 2020; Carignan et al., 2020; Giacomelli et al., 2020; Hopkins, Surda, & Kumar, 2020; Hopkins, Surda, Whitehead, et al., 2020; Iravani et al., 2020; Kaye et al., 2020; Klopfenstein et al., 2020; Kosugi et al., 2020; Lechien, Cabaraux, & Chiesa‐Estomba et al., 2020a, 2020b; Lechien et al., 2020; Lee et al., 2020; Levinson et al.,^2020^;Mao et al., 2020;Moein et al., 2020; Spinato et al., 2020; Vaira et al., 2020, 2020; Wee et al., 2020; Yan et al., 2020) High prevalence of these symptoms was reported in both studies with an objective assessment of taste and smell and self‐reported symptom questionnaires (Beltrán‐Corbellini et al., 2020; Bénézit et al., 2020; Boscolo‐Rizzo et al., 2020; Carignan et al., 2020; Giacomelli et al., 2020; Hopkins, Surda, & Kumar, 2020; Hopkins, Surda, Whitehead, et al., 2020; Iravani et al., 2020; Kaye et al., 2020; Klopfenstein et al., 2020; Kosugi et al., 2020; Lechien, Cabaraux, & Chiesa‐Estomba et al., 2020a, 2020b; Lechien et al., 2020; Lee et al., 2020; Levinson et al., 2020; Mao et al., 2020; Moein et al., 2020; Spinato et al., 2020; Vaira et al., 2020, 2020; Wee et al., 2020; Yan et al., 2020) OTD was found to be significantly higher in females and in younger patients (Beltrán‐Corbellini et al., 2020; Bénézit et al., 2020; Boscolo‐Rizzo et al., 2020; Carignan et al., 2020; Giacomelli et al., 2020; Hopkins, Surda, & Kumar, 2020; Hopkins, Surda, Whitehead, et al., 2020; Iravani et al., 2020; Kaye et al., 2020; Klopfenstein et al., 2020; Kosugi et al., 2020; Lechien, Cabaraux, & Chiesa‐Estomba et al., 2020a, 2020b; Lechien et al., 2020; Lee et al., 2020; Levinson et al., 2020; Mao et al., 2020; Moein et al., 2020; Spinato et al., 2020; Vaira et al., 2020, 2020; Wee et al., 2020; Yan et al., 2020). Indeed, neurological manifestations of COVID‐19 such as Guillain–Barré syndrome (GBS) in young people are of great concern (Ahmed et al., 2020). However, so far only a few cases of COVID‐19 associated with GBS have been reported (Webb, Wallace, & Martin‐Lopez, 2020). GBS can rarely present with taste dysfunction (Kogan, Mednick, & Dolgovina, 2011). Although currently there is no literature on the prevalence of OTD in COVID‐19‐positive patients with GBS, we speculate that increased prevalence of taste dysfunction in GBS patients may raise the suspicion of the infection.

Postviral anosmia has been reported to account for 40% of the anosmia cases and one of the leading causes of anosmia in adults (Hummel et al., 2017). There is variability in OTD dysfunction among patients, and its association with the severity of COVID‐19 infection is currently uncertain. However, a significant correlation between the duration of olfactory and gustatory symptoms and severe COVID‐19 infection has been reported (Vaira et al., 2020). Therefore, hospitalized patients especially younger patients with OTD should be monitored carefully. In terms of other COVID‐19 symptoms, fever and cough were the most common, which is consistent with already published medical literature (Boscolo‐Rizzo et al., 2020; Carignan et al., 2020; Giacomelli et al., 2020; Iravani et al., 2020; Klopfenstein et al., 2020; Kosugi et al., 2020; Lechien, Cabaraux, & Chiesa‐Estomba et al., 2020a, 2020b; Lechien et al., 2020; Lee et al., 2020; Lovato & Filippis, 2020; Mao et al., 2020; Moein et al., 2020; Spinato et al., 2020; Vaira et al., 2020; Wee et al., 2020; Yan et al., 2020).

The presence of anosmia and dysgeusia could help neurologists and otolaryngologists identify COVID‐19 cases early, allowing prompt treatment and reduction in infection transmission. It is possible that symptoms of anosmia and dysgeusia can be used as an effective screening tool and aid diagnosis of COVID‐19. Zayet et al. suggest that the positive predictive value (PPV) for anosmia is 77%, dysgeusia 77% and a combination of anosmia plus dysgeusia 83% for a positive SARS‐CoV‐2 real time‐polymerase chain reaction (RT‐PCR) test on a nasopharyngeal sample (Zayet et al., 2020). However, a key limitation of Zayet et al.'s study is that although RT‐PCR on a nasopharyngeal sample is specific for COVID‐19, it has a sensitivity of 56%‐83% and may be an inaccurate test to diagnose COVID‐19 (AAO‐HNS: Anosmia, Hyposmia; Kokkinakis, Selby, Favrat, Genton, & Cornuz, 2020). Therefore, some patients with anosmia and dysgeusia that tested negative for COVID‐19 may be infected with the disease. Further research is required to confidently determine the utility of OTD dysfunction as an effective screening tool for COVID‐19 infection.

### 3.9 Mechanisms underlying olfactory and gustatory function in COVID‐19 patients

**Figure 2:**
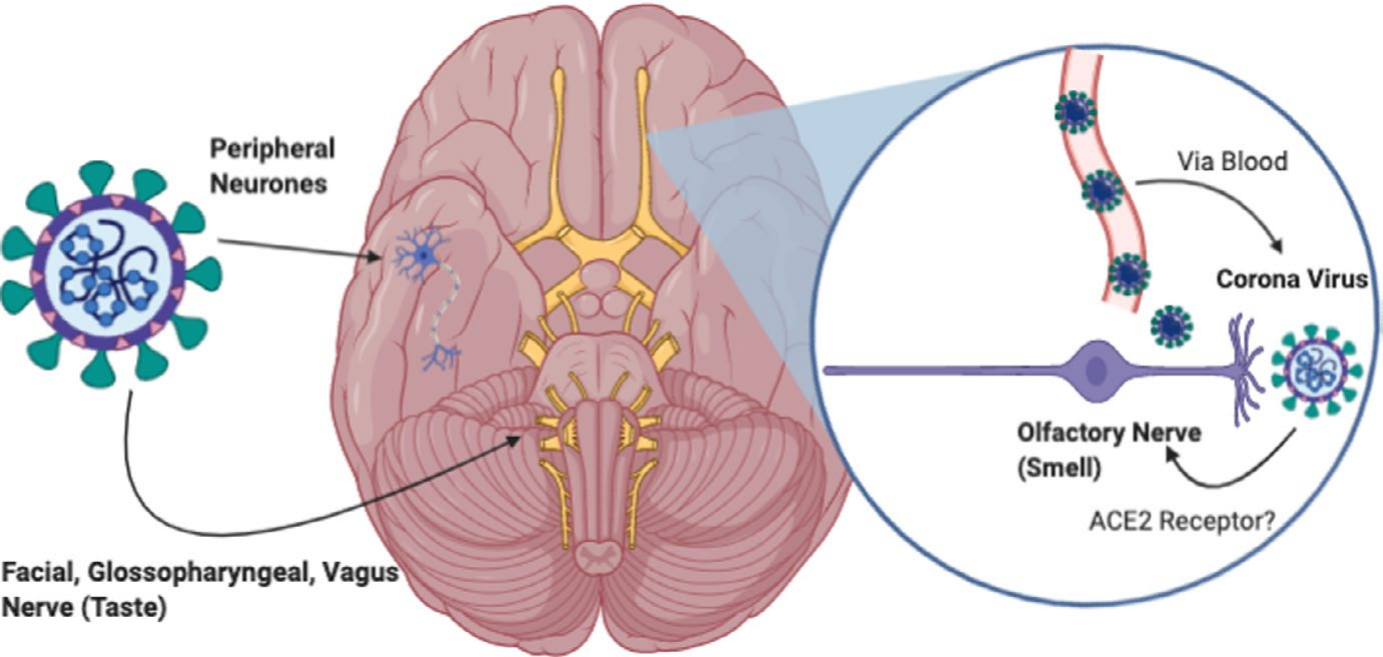
Possible mechanism of interaction between SARs‐CoV‐2 and the cranial nerves. SARS‐CoV‐2 can potentially interact with the cranial nerves (olfactory, vagus, facial, and glossopharyngeal) via the angiotensin‐converting enzyme receptor‐2 (ACE2) leading to olfactory taste dysfunction. SARS‐CoV‐2 can also cause damage to the peripheral nerves leading to several other neurological manifestations. Angiotensin‐converting enzyme 2; ACE2, Original Illustration created using BioRender

Anosmia associated with COVID‐19 has been well documented by the literature (Beltrán‐Corbellini et al., 2020; Bénézit et al., 2020; Boscolo‐Rizzo et al., 2020; Carignan et al., 2020; Giacomelli et al., 2020; Hopkins, Surda, & Kumar, 2020; Hopkins, Surda, Whitehead, et al., 2020; Iravani et al., 2020; Kaye et al., 2020; Klopfenstein et al., 2020; Kosugi et al., 2020; Lechien, Cabaraux, & Chiesa‐Estomba et al., 2020a, 2020b; Lechien et al., 2020; Levinson et al.,^2020^;Lovato &Filippis, 2020;Mao et al., 2020;Moein et al., 2020;Spinato et al., 2020; Vaira et al., 2020, 2020; Wee et al., 2020; Whitcroft & Hummel, 2020; Yan et al., 2020) Netland et al. reported that SARS‐CoV can cause neuronal death in mice by invading the brain via the nose, which is close to the olfactory epithelium (Netland, Meyerholz, Moore, Cassell, & Perlman, 2008). Furthermore, the human coronavirus 229E has been isolated in nasal discharge from a patient with postviral olfactory dysfunction (Suzuki et al., 2007). It has been proposed that SARS‐CoV‐2 gains entry into the central nervous systems via several different ways (Ahmed et al., 2020). One of the proposed mechanisms is dissemination and spread from the cribriform plate which is in close contact to the olfactory bulb; this mechanism is postulated to be the underlying cause of olfactory dysfunction in patients (Baig, Khaleeq, Ali, & Syeda, 2020). Furthermore, the general presence of the virus in the circulation can lead to systematic dissemination, allowing the virus to enter the cerebral circulation (Ahmed et al., 2020). The brain has been reported to express ACE2 receptors mainly on glial cells, neurons, and brain vasculature, making these cells susceptible to attacks by the SAR‐CoV‐2 (Ahmed et al., 2020; Turner, Hiscox, & Hooper, 2004). It is well‐established that SARS‐CoV‐2 can exploit the angiotensin‐converting enzyme 2 (ACE2) receptor to gain entry into the cells (Ahmed et al., 2020). Viral interaction with the expression of the ACE2 receptor in neurones can result in significant damage to the neurones without substantial association inflammation previously observed with SARS‐CoV infection (Ahmed et al., 2020; Netland et al., 2008; Wrapp et al., 2020). Therefore, it can be postulated that SARS‐CoV‐2 can cause neuronal damage via the ACE2 receptor leading to OTD (Figure 2) (Ahmed et al., 2020). Binding of the virus to the ACE2 receptors may cause endothelial dysfunction and also lead to serious consequences such as cerebral hemorrhage via unknown mechanisms (Ahmed et al., 2020). Another mechanism by which the neurotropic SARs‐CoV‐2 can disseminate through the central nervous system is by anterograde and retrograde transport with the aid of motor proteins such as kinesins and dynein via sensory and motor nerve endings, the afferent nerve endings from the vagus nerve from the lungs are especially implicated (Ahmed et al., 2020; Li, Bai, & Hashikawa, 2020; Swanson & McGavern, 2015). Through this mechanism, SARS‐CoV‐2 could potentially cause gustatory dysfunction. Besides, other ways in which SARS‐CoV‐2 can cause neurological damage leading to OTD is through a surge of inflammatory cytokines leading to cytokine storm syndrome (Ahmed et al., 2020; Wan, Yi et al., 2020).

**Figure 3:**
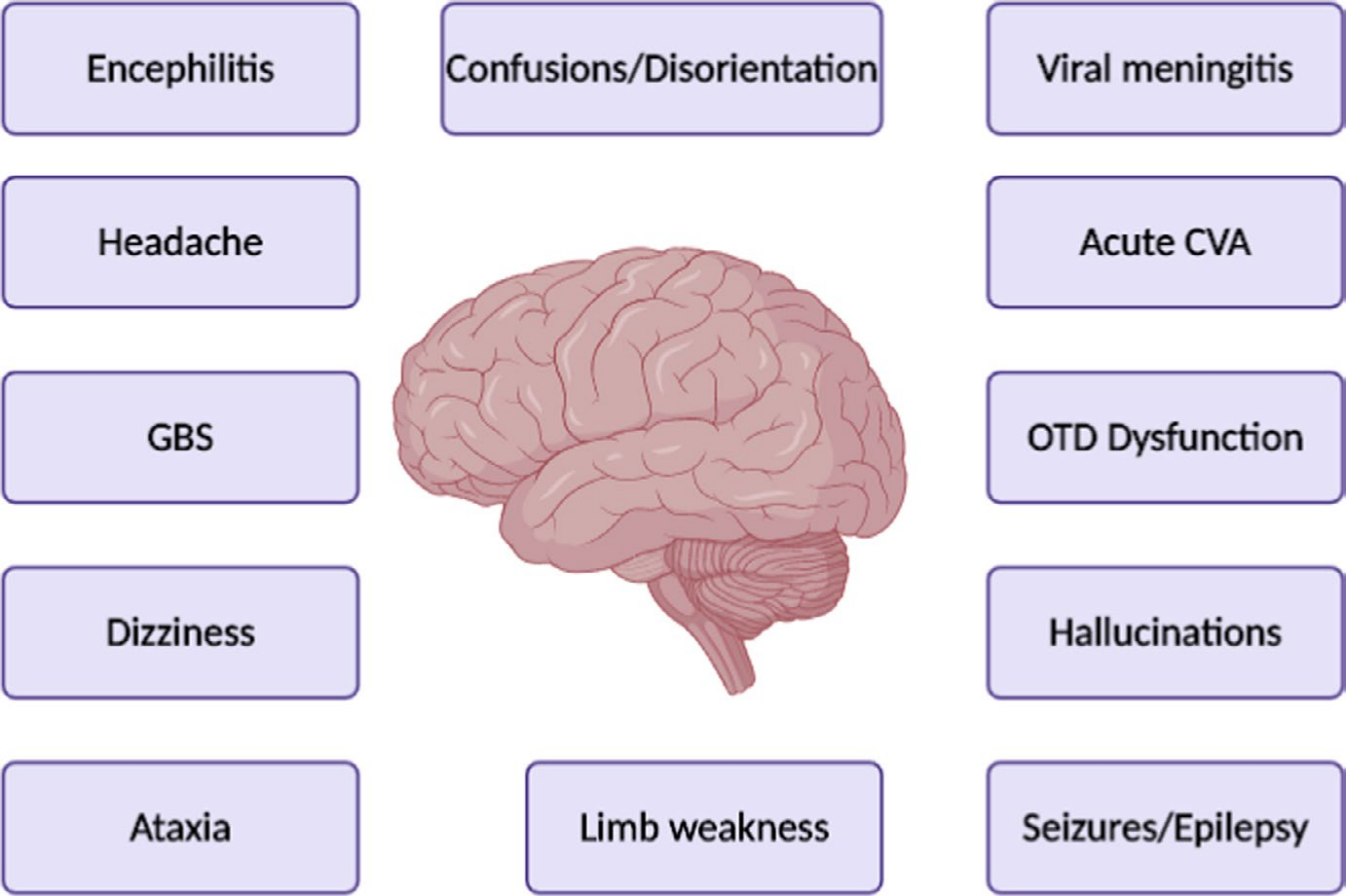
Neurological manifestations of COVID‐19. Guillain–Barré syndrome; GBS, Cerebrovascular Accidents; CVA, Olfactory and taste dysfunction; OTD Original Illustration created using BioRender

COVID‐19 has also been reported to cause other neurological manifestations such as seizures, headache, and dizziness (Figure 3) (Ahmed et al., 2020; Whittaker, Anson, & Harky, 2020). Table 4 shows a summary of neurological manifestations of COVID‐19 reported by literature (Abdelnour, Abdalla, & Babiker, 2020; Alberti et al., 2020; Bernard‐Valnet et al., 2020; Camdessanche et al., 2020; Chen, Zhou, et al., 2020; Chen, Wu, et al., 2020; Coen et al., 2020; Duong, Xu, & Liu, 2020; Guan et al., 2020; Haddadi, Ghasemian, & Shafizad, 2020; Huang et al., 2020; Kaya, Kara, Akinci, & Kocaman, 2020; Klok et al., 2020; Li et al., 2020; Lodigiani et al., 2020; Mao et al., 2020; Moriguchi et al., 2020; Oxley et al., 2020; Padroni et al., 2020; Poyiadji et al., 2020; Sedaghat & Karimi, 2020; Sharifi‐Razavi, Karimi, & Rouhani, 2020; Toscano et al., 2020; Virani et al., 2020; Wan, Xiang et al., 2020; Wan, Cao, Fang, Wang, & Huang, 2020; Wang, Hu et al., 2020; Wang, Yang, Li, Wen, & Zhang, 2020; Yang et al., 2020; Ye, Ren, & Lv, 2020; Zhao, Shen, Zhou, Liu, & Chen, 2020; Zhou et al., 2020). It is unclear whether symptoms of OTD alone can be used to diagnose COVID‐19. However, during this pandemic, COVID‐19 should be considered an important differential diagnosis for patients presenting with OTD dysfunction. Nevertheless, the presence of these symptoms alongside thorough clinical examination, microbiology tests, and the use of diagnostic imaging techniques can help to eliminate differentials and aid the diagnosis of COVID‐19. Chemosensory assessment and treatments used for postviral OTD may also be potentially beneficial in COVID‐19 patients (Whitcroft & Hummel, 2020).

**Table 4:** Neurological manifestations of COVID‐19

Author	Type of publication	Number of COVID−19‐Positive Patients	Results
Neuropathy			
Abdelnour et al. (2020)	Case Report	1	Bilateral lower limb weakness in 69‐year‐old male
Wan et al., 2020	Case Report	1	Bell's palsy in a 65‐year‐old female.
Stroke			
Klok et al. (2020)	Retrospective observational study	184	1.6% develop an ischemic or hemorrhagic stroke
Lodigiani et al. (2020)	Retrospective observational study	338	2.5% developed an ischemic or hemorrhagic stroke
Mao et al. (2020)	Retrospective observational study	214	2.8% developed an ischemic or hemorrhagic stroke
Li et al. (2020a)	Retrospective observational study	219	6.0% developed an ischemic or hemorrhagic stroke
Sharifi‐Razavi et al. (2020)	Case Report	1	Intracerebral hemorrhage in 79‐year‐old male with
Haddadi et al. (2020)	Case Report	1	Intracerebral hemorrhage in 54‐year‐old female
Oxley et al. (2020)	Case Report	5	Ischemic stroke in patients younger than 50 years of age
Guillain–Barré Syndrome (GBS)			
Alberti et al. (2020)	Case report	1	71‐year‐old male patient presented with symptoms of subacute onset of paresthesia at limb extremities, distal weakness rapidly evolving to severe, flaccid tetra paresis over 3 days consistent with the diagnosis of GBS
Toscano et al. (2020)	Case report	5	Symptoms of GBS including lower limb weakness and paresthesia in 4 patients and facial diplegia followed by ataxia and paresthesia in 1 patient. Generalized, flaccid tetra paresis/tetraplegia over a period of 36 hr to 4 days in 4 patients.
Coen et al. (2020)	Case report	1	Male patient in his 70’s presented with paraparesis, distal allodynia, difficulties in voiding and constipation preceding symptoms of myalgia, fatigue, and a dry cough.
Camdessanche et al. (2020)	Case report	1	Hospitalized 64‐year‐old man COVID−19‐positive patients developed paresthesia in feet and hands and later flaccid severe tetra paresis. Patient was diagnosed with GBS on the basis of neurological examination and investigations.
Virani et al. (2020)	Case report	1	54‐year‐old male presented with numbness and weakness and was diagnosed with GBS and COVID−19 positive.
Zhao et al. (2020)	Case Report	1	61‐year‐old female presented with weakness in both legs and severe fatigue, progressing over a day, was diagnosed with GBS. Later, she developed respiratory symptoms of COVID−19
Padroni et al. (2020)	Case Report	1	70‐year‐old female was presented to the emergency department complaining of asthenia, hands and feet paresthesia and gait difficulties progressing within 1 day. Diagnosed with GBS, later developed respiratory symptoms of COVID−19
Sedaghat et al. (2020)	Case Report	1	65‐years‐ old male patient presented to emergency department, with symptoms of acute progressive symmetric ascending quadriparesis. 2 weeks prior to onset of neurological symptoms, he was diagnosed with COVID−19.
Headache			
Wan et al., 2020	Case series	135	33% of patients reported a headache
Wang et al. (2020)	Case series	138	7% of patients reported a headache
Wang et al. (2020)	Retrospective review	69	14% of patients reported a headache
Yang et al. (2020)	Retrospective review	52	6% of patients reported a headache
Mao et al. (2020)	Retrospective study	214	13% patients reported headache
Chen, Wu, et al. (2020)	Retrospective study	99	8% patients reported headache
Dizziness			
Mao et al. (2020)	Retrospective review	214	17% of patients reported dizziness
Chen, Wu, et al. (2020)	Retrospective review	113	8% of patients reported dizziness
Wang et al. (2020)	Retrospective review	138	6.5% of patients reported dizziness
Myalgia			
Wang et al. (2020)	Retrospective review	138	34.8% of patients reported myalgia
Zhou et al. (2020)	Retrospective review	191	15.2% of patients reported myalgia
Chen, Wu, et al. (2020)	Retrospective review	99	11% of patients reported myalgia
Huang et al. (2020)	Retrospective review	41	44% of patients reported myalgia or arthralgia
Guan et al. (2020)	Retrospective review	1,099	15% of patients reported myalgia or arthralgia
Encephalitis			
Ye et al. (2020)	Case report	1	Fever, shortness of breath and myalgia with diminished consciousness
Bernard‐Valnet et al. (2020)	Case report	2	1 patient developed tonic‐clonic seizures and lumbar puncture consistent with viral encephalitis 1 other patient developed intense headache with confirmed SARS‐CoV−2 swab
Poyiadji et al. (2020)	Case report	1	58‐year‐old female presented with symptoms of COVID−19 and altered mental state.
Confusion			
Chen, Wu, et al. (2020)	Retrospective review	99	9.10% of patients reported confusions
Kaya et al. (2020)	Case report	1	Patient reported confusion and visual agnosia
Meningitis			
Moriguchi et al. (2020)	Case report	1	24‐year‐old male developed symptoms of meningitis/encephalitis
Status epilepticus/Seizures			
Mao et al. (2020)	Retrospective review	214	0.5% of patients presented with seizures
Doug et al. (2020)	Case Report	1	41‐year‐old female presented with headache, confusion fever, and new‐onset seizure
Moriguchi et al. (2020)	Case Report	1	24‐year‐old male bought to the emergency department due to convulsions with impaired consciousness. The patient had symptoms and imaging was consistent with a diagnosis of meningitis and patient was COVID−19 positive.

### 3.10 Limitations

Our review, only focussed on COVID‐19‐positive patients, however, these symptoms may also be present in patients that were COVID‐19‐negative. Therefore, to fully establish the diagnostic value of OTD comparison of symptoms between both COVID‐19‐positive and COVID‐19‐negative patients should be investigated. The risk versus benefit of researching COVID‐19 and OTD also needs to be highlighted. In studies with an objective assessment of OTD, there is possible risk of infection transmission due to physician–patient contact. Although telephone interviews and online surveys can help to minimize this risk, there is a potential bias associated with subjective assessment of symptoms. Another key limitation of our review is that we did not consider the limitations of the method of assessment of OTD in the studies. Studies used either individualized online surveys or specific tests such as CCCRC and SNOT‐22. The differences between the questionnaires and reliability of the objective tests in reporting OTD were not taken into consideration. Currently, in clinical practice, subjective assessment of chemosensory function is relied upon as there is a limited correlation with objective measures. Furthermore, key confounding factors such as underlying respiratory disease or hay fever were not discussed. Moreover, the follow‐up duration of the recovery of patients with OTD is not well documented. Finally, it is possible that some studies would have rushed to publish presenting incomplete data.

## 4 IMPLICATIONS FOR FURTHER RESEARCH

Currently, the literature on COVID‐19 and OTD is limited and is mainly confined to reports of self‐reported symptoms. Further studies with large cohort sizes and global collaborations with an objective assessment of OTD are required to fully establish the merit of these symptoms in the diagnosis of COVID‐19. OTD can also be present in non‐COVID‐19 patients (Kosugi et al., 2020). Kosugi et al. reported OTD in patients which subsequently tested negative for COVID‐19 (Kosugi et al., 2020). Future studies with larger cohort sizes comparing the difference in duration and prevalence of OTD in COVID‐19‐positive patients compared to non‐COVID‐19 patients would help elucidate the true diagnostic value of these symptoms in COVID‐19. OTD tends to significantly affect patients who are female and from younger age groups; however, the reasons are unknown. Furthermore, there is uncertainty regarding the utility of these symptoms as a predictor of severe COVID‐19 infection. Therefore, further research is required to clarify the association between OTD and the severity of COVID‐19 infection. The pathophysiology of OTD in COVID‐19 remains to be determined.

## 5 CONCLUSION

Clinical examination of the olfactory nerve is often neglected in routine clinical practice. The COVID‐19 pandemic has highlighted the importance of this much forgotten cranial nerve. COVID‐19 patients frequently report symptoms of anosmia and dysgeusia, and therefore, these symptoms should raise a high index of suspicion for COVID‐19 infection especially in young and female patients. The presence of these symptoms alongside objective clinical assessment would help to make a diagnosis. Further research is warranted as currently both the performance of these symptoms as predictors of COVID‐19 infection and their diagnostic value is uncertain.

## DISCLOSURES

All authors have no conflicts of interest to declare.

## AUTHOR CONTRIBUTIONS

Syeda Anum Zahra planned and drafted the manuscript, created figures, and is responsible for the final draft of the manuscript. Kiran Pillai and Sashini Iddawela researched and helped with drafting and formatting the final manuscript. Rozina Choudhury helped with drafting and formatting the final manuscript. Amer Harky provided supervision and edited the final manuscript.

### Peer Review

The peer review history for this article is available at https://publons.com/publon/10.1002/brb3.1839.
